# Statistical analysis plan for the PRO B study: open-label, superiority randomised controlled trial of alarm-based patient-reported outcome monitoring in patients with metastatic breast cancer

**DOI:** 10.1186/s13063-024-08025-9

**Published:** 2024-03-07

**Authors:** Pimrapat Gebert, Maria Margarete Karsten, Anna Maria Hage, Adam David Dordevic, Ulrike Grittner

**Affiliations:** 1 Berlin Institute of Health at Charité-Universitätsmedizin Berlin, Charitéplatz 1, Berlin, 10117 Germany; 2 Institute of Biometry and Clinical Epidemiology, Charité-Universitätsmedizin Berlin, Charitéplatz 1, Berlin, 10117 Germany; 3 Department of Gynecology with Breast Center, Charité – Universitätsmedizin Berlin, Charitéplatz 1, Berlin, 10117 Germany

**Keywords:** Metastatic breast cancer, Patient-reported outcomes, Patient monitoring, Quality of life, Digital health, ePROs, Statistical analysis plan

## Abstract

**Background:**

With an increasing collection of patient-reported outcomes (PROs) to measure health-related quality of life (HRQoL) in oncological patients, there is still a lack of standardised strategies on how to interpret and use these data in patient care. Prior research has shown support for the use of digital PRO monitoring together with alarm systems to notify clinicians when the PRO values are deteriorating. This system has demonstrated advantages in improving HRQoL and increasing survival rates among oncology patients. Hence, we designed the PRO B study, a superiority multi-centre randomised controlled trial, to investigate the effects of alarm-based monitoring in metastatic breast cancer patients in Germany. The study protocol for the PRO B study was published in September 2021, and this manuscript describes a formal statistical analysis plan (SAP) for the PRO B study to improve the transparency and quality of this trial.

**Methods and design:**

The trial aimed to recruit 1000 patients with metastatic breast cancer. However, as of the completion of recruitment on June 15, 2023, we have successfully enrolled 924 patients from 52 breast cancer centres. Patients were 1:1 stratified randomised to the intervention and control groups. App-based PRO questionnaires are sent weekly to the intervention group and every 3 months to the control group. Only patients in the intervention group trigger an alarm if their PRO scores deteriorate, and they are subsequently contacted by the local care team within 48 h. The primary outcome is the fatigue score at 6 months, and secondary outcomes are other HRQoL and overall survival. Evaluation of the superiority of the intervention will be done using a linear mixed model with random intercepts for study centres.

**Conclusion:**

This detailed SAP defines the main components of the statistical analysis for the PRO B study to assist the statistician and prevent bias in selecting analysis and reporting findings. Version 1 of the SAP was finalised on January 18, 2024.

**Trial registration:**

DRKS (German Clinical Trials Register) DRKS00024015. Registered on February 15, 2021.

**Supplementary Information:**

The online version contains supplementary material available at 10.1186/s13063-024-08025-9.

## Background

Metastatic breast cancer (MBC) is a clinical challenge that requires a multidimensional approach as it affects the patient’s overall survival and quality of life (QoL), among others. Nowadays, incorporating the digital measurement of patient-reported outcomes (PROs) assists patient monitoring to track, e.g., symptoms, QoL, or adherence to medication in real time. Regular alarm-based PRO monitoring in metastatic cancer patients has shown benefits in terms of improved QoL, reduced emergency department visits, and better overall survival [1–3]. Therefore, alarm-based PRO monitoring assists in the detection of a deterioration of health-related QoL (HRQoL) and symptoms and might facilitate early symptom management and treatment adaptations that might, in turn, shorten the waiting time for effective therapy and prolong the patient’s overall survival.

The PRO B study is designed to investigate whether alarm-based PRO monitoring is feasible in the German medical care system and if its benefits are reproducible in breast cancer care in Germany. In the multicentric randomised-controlled study all over Germany with metastatic breast cancer patients, 52 centres recruited 924 of the 1000 prespecified patients. The patients were 1:1 stratified randomised to an intervention and control group and subsequently assigned app-based questionnaires at the time of inclusion (baseline). The intervention group is then assessed with a weekly PRO questionnaire that is linked to an alarm system that alerts the local care team in case of worsening PRO scores. Their PRO values are graphically displayed for the care team during the study, and worsened PRO domains are highlighted in red. When the digital PRO tool activates an alarm, the local care team receives an email and is required to reach out to the patient within 48 h to address the deterioration and document the communication. In contrast to that, the control group is assigned questionnaires only every 3 months; their PRO scores are not displayed for the care team and do not trigger alarms with worsened PRO scores.

The PRO B study has been registered with DRKS (German Clinical Trials Register) DRKS00024015 on February 15, 2021, and its protocol (version 2.2, dated April 2, 2021) has been published [4]. In this paper, we provide our detailed statistical analysis plan (SAP) for the PRO B study, following the published SAP guidelines [5]. This SAP solely addresses the objectives indicated in this article; long-term results and health economic analysis are not provided.

### Primary hypothesis

The main hypothesis of the PRO B study is that patients who receive weekly electronic PRO monitoring with alarm generation will have significantly lower fatigue scores after 6 months when compared to the patients who receive a PRO survey every 3 months.

### Secondary hypotheses

In addition to the primary hypothesis, the study’s secondary hypotheses aim to assess the effectiveness of the intervention with regard to other HRQoL and survival outcomes compared to the control group. These secondary hypotheses are as follows:

- The intervention group will have fewer emergency room visits and hospitalisations compared to the control group at 6 and 12 months.
- The physical functioning scores at 6 and 12 months will be higher in the intervention group compared to the control group.
- The fatigue score at 12 months will be lower in the intervention group compared to the control group.
- In a subgroup of patients with visceral metastases, the intervention group will have a better overall survival compared to the control group at 12 months.
- In a subgroup of patients with triple-negative breast cancer, the intervention group will demonstrate better overall survival compared to the control group at 12 months.
- Scores of HRQoL at 6 and 12 months will be higher in the intervention group compared to the control group.
- Patients in the intervention group will have a shorter time to first systemic therapy change than patients in the control group due to early detection of therapy intolerance or disease progression.

### Detailed trial design

PRO B is an open-label, multi-centre superiority randomised controlled trial in patients with metastatic breast cancer (MBC). After providing written informed consent and downloading the smartphone application, patients are randomised to the study group in a 1:1 ratio stratified based on the following strata: breast cancer centres, types of remote metastases (bone, lymph node, visceral, brain, or multiple sites), and hormone receptor (HR) status (HR + or HR −), using adaptive randomisation from the secuTrial® electronic randomisation software administered by the Clinical Trial Office (CTO) of Charité—Universitätsmedizin Berlin [4]. Patients eligible for PRO B must be female, aged over 18, undergoing drug treatment for MBC with a life expectancy exceeding 3 months, and receiving care at a participating breast cancer centre. Additionally, they should have internet access through a smartphone to download the study app, an ECOG performance status of 0 to 2, and a willingness to engage in a weekly digital PRO survey. Patients not receiving active anti-cancer treatment (comfort care) or failing to meet the outlined inclusion criteria are not eligible for the PRO B study.

The PRO survey is based on the patient-reported outcome measures developed by the Quality of Life Group of the European Organisation for Research and Treatment of Cancer (EORTC). The EORTC QLQ-C30, its core instrument, includes five functional dimensions (physical function, role function, emotional function, cognitive function, and social function) and nine symptom scales (fatigue, nausea, pain, dyspnea, insomnia, appetite loss, constipation, diarrhoea, and financial difficulties) [6]. To assess these domains, we extended the QLQ-C30 with items from the EORTC item library in order to cover more item content and extend measurement precision. For example, we extended symptom scales to contain two items at minimum. Scoring of these tailored short forms was performed on the IRT models of the EORTC Computer Adaptive Testing (CAT) Core Itembanks [7]. This yields scores on a standardised T-metric with a mean of 50 and a standard deviation (SD) of 10 in the general population. This scoring system is unlike the scoring of the EORTC QLQ-C30, which presents scores on a scale of 0–100 [8] and is more informative than simple C30 scores [9].

Details of the study design are described in a separate protocol article [4].

### Sample size calculation

Based on the published protocol [4], the trial aimed to enroll 1000 patients (500 cases per group) with a power of 80.65% and a two-sided significance level of 5% to detect a minimal clinically relevant effect of 5 scores mean difference on the fatigue scale of the EORTC QLQ-C30 with a common standard deviation of 25 scores (a minimum standardised effect size of 0.2) [10, 11]. The recruiting period of 12 months was extended by 4 months since the desired sample size could not be obtained in the initially scheduled recruitment period. At the end, the study recruited 924 MBC patients (461 for the control group and 463 for the intervention group).

### Current status of trial

Enrollment for PRO B began on May 17, 2021. The study extended the recruitment time and ended on June 15, 2023. The intervention and data collection are ongoing, and the last follow-up for the final participants is currently planned for February 15, 2024.

### Outcomes

Study outcomes are defined in Table 1. All time frames start at the date of randomisation. In case a patient in the intervention group did not complete the weekly PRO survey at the scheduled time, we will use the PRO scores from the week before or after (+ / − 1 week). For example, if a patient misses the 26-week PRO B survey (6 months), but we have PRO data from 25 or 27 weeks, we will substitute the 26-week PRO data with the scores from either 25 or 27 weeks, depending on availability. If all assessments (prior and post-missing time points) are observed, we will use the PRO value before the missing time point.

**Table 1:** Primary and secondary outcomes

Outcomes	Description
**Primary outcome**	
**Fatigue score**	Standardised T-metric score at 6 months. Higher scores represent more fatigue burden.
**Secondary outcomes**	
**Need of hospitalisations and emergency room visits**	A cumulative number of hospital admissions and number of emergency room visits at 6 and 12 months.
**Physical functioning score**	Standardised T-metric score at 6 and 12 months. Higher scores indicate higher levels of functioning.
**Fatigue score**	Standardised T-metric score at 12 months. Higher scores represent more fatigue burden.
**Overall survival**	Time to death (in months).
**HRQoL score**	Standardised T-metric score at 6 and 12 months. Higher scores indicate higher levels of HRQoL.
**Time to first systemic therapy change**	Time to first systemic therapy change (in months) after randomisation.

### Statistical methods

The statistical analyses for the specified aims will follow the statistical principles described below. Long-term outcomes and health economic analysis are not specified in this SAP and will be described separately in another manuscript.

### General principles

We will conduct an analysis of the PRO B study data using the modified intention-to-treat (mITT) principle. In this approach, the ITT population will include all randomised participants who have responded to at least one PRO survey. However, patients who have not completed any PRO surveys during the study period will be excluded from the analysis. This definition of the mITT differs from the study protocol [4], excluding the fact that participants now exhibit complete non-response to the PRO surveys. In such instances, they are regarded as not having received any impact from the intervention.

The primary endpoint will be tested at a two-sided 0.05 significance level, and 95% confidence intervals are calculated using robust estimation methods. Other secondary endpoints will be analysed exploratorily. No adjustment of *p*-values for multiple testing will be made for secondary endpoint analyses. Stratification variables of the randomisation (types of metastases and hormone receptor status) will be included as fixed factors in all statistical models.

### Potential confounding factors

Potential confounding factors that are considered to adjust for all the analyses are listed below:

- Age at randomisation (years)
- Type of metastases (bone, lymph node, visceral, brain, or multiple sites)
- Hormone receptor status (HR + or HR −)
- Types of systemic therapy at randomisation (endocrine, chemotherapy, anti-Her2 therapy, immunotherapy, targeted therapy, and others)
- ECOG status at enrolment (ECOG 0, 1, 2)
- Changing alarm criteria (before and after September 1, 2022)
- Socio-economic and anamnesis parameters
  - ◦ Marital status (multiple categories)
  - ◦ Having dependents in need of care or underage children (≤ 14 years) in the household (yes or no)
  - ◦ Educational status (multiple categories, according to ISCED [12])
  - ◦ Employment status (yes or no)
  - ◦ Income (multiple categories)
  - ◦ Migration background (yes or no)
  - ◦ National Cancer Institute (NCI) comorbidity index (numeric)

### Socio-economic and anamnesis baseline characteristics

The socio-economic and anamnesis baseline characteristics are listed in the Supplementary Table S1, and the findings will be presented using descriptive statistics. The participant count (*n*) is specified for each group. Continuous variables will be presented with mean and standard deviation (SD), median, interquartile range (IQR), minimum, and maximum based on the distribution. Categorical data will be represented by absolute and relative frequencies for each category. The NCI comorbidity index using weights specific for breast cancer patients will be calculated [13].

### Analysis of the primary outcome

#### Fatigue scores at 6 months

We will use a linear mixed-effects regression model accounting for study centres as a random intercept. In line with the primary hypothesis of the superiority of the intervention compared to the control group, the fatigue score at 6 months will be compared between the intervention and the control groups. In addition to the above potential confounding factors, the analysis will be adjusted for the baseline fatigue score. The superiority hypothesis to be tested can be stated as:

$$\begin{array}{ccc}H_0:\;\mu_{\mathrm{IG}}\mathit-\mathit\;\mu_{\mathit{CG}}=\;0\;&\mathrm{versus}&H_1:\;\mu_{\mathrm{IG}}\;-\;\mu_{\mathrm{CG}}\;\neq\;0\end{array}$$

where ${\mu }_{{\text{IG}}}$ and ${\mu }_{{\text{CG}}}$ are the means of the fatigue score at 6 months for the intervention group (IG) and control group (CG). The hypothesis will be tested in the modified intention-to-treat population. The effect estimate (adjusted mean group difference) will be reported with the corresponding two-sided 95% CI.

**Fig. 1:**
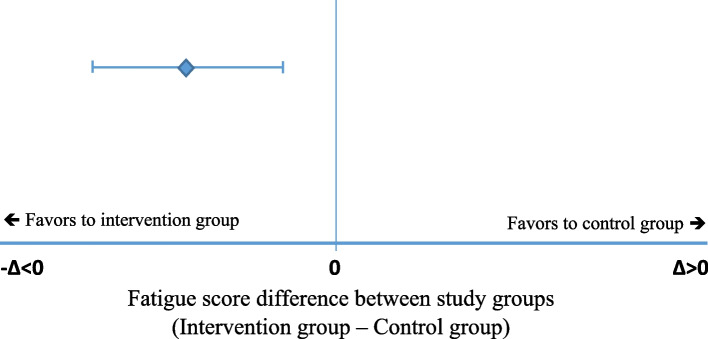
Superiority concept for the comparison of fatigue scores between study groups

Figure 1 presents a virtual example of an intervention effect. Superiority will be considered if the fatigue score in the intervention group is lower than that in the control group. Therefore, the mean difference of the fatigue score between the intervention and control groups (point estimate) and the 95%CI of the mean difference are lower than zero and do not include zero (solid vertical line).

### Analysis of the secondary outcomes

#### The effect of intervention on emergency room visits and hospitalisations

The number of hospital admissions and emergency room visits will be counted per patient and divided by the duration of observation in month-unit (hospital admission/person-month or emergency room visit/person-month). We will report incidence rates per group as descriptive statistics. To investigate if the intervention has an effect on the need for hospital admissions and emergency room visits, we will estimate the incidence rate ratio (IRR) and 95%CI by using a mixed-effects Poisson model with a log link function and random intercepts for study centres. We will check overdispersion by comparing the models between the Poisson and negative binomial regression models using the likelihood ratio test.

#### Overall survival

Time-to-death will be defined as the time lag between the date of randomisation and the date of death or censored at the last follow-up date or the date of the study’s end. If only the year and month of the patient’s death are known but the precise date remains uncertain, the 15th of the month of death will be applied. The survival distribution of OS will be estimated using the Kaplan–Meier method. The results will be visually presented through Kaplan–Meier curves for each study group, illustrating the number of patients at risk at each time point. Median OS and 95%CI will be provided for each group. The survival probabilities at 3, 6, and 12 months and 95%CI will be summarised by the study arm. A Cox proportional hazards model with a random shared frailty term [14] will be performed to compare OS between study groups, accounting for study centres as a random intercept. The hazard ratio (HR) with a two-sided 95%CI will be presented.

#### The effect of intervention on physical functioning and health-related quality of life

We will use linear mixed models to compare the physical functioning scores and HRQoL between study groups. Interaction terms for the study time points (e.g., 3-month, 6-month, 12-month) and group will be included to capture differential changes over time. The study centres and patients will be set as random intercepts (three-level model). Adjusted coefficients representing the adjusted mean difference between groups at each time point and 95%CI will be reported and visually depicted through plots.

#### Time to first systemic therapy change

Time to first systemic therapy change will be defined as the time lag between the date of randomisation and the date of the first systemic therapy change, and censored will be considered at the last follow-up date or the date of the study end. Death as an event occurring before changing the first systemic therapy will be considered a competing event. The cumulative incidence of systemic therapy changes will be presented. We will estimate the sub-distribution hazards ratio (SHR) and its 95%CI using Fine and Gray’s proportional sub-distribution hazards models, assuming that death is a competing risk and a shared frailty effect to account for study centres. We will apply the Cox proportional hazards model in case the competing event is less than 10% or no more than the proportion of the first systemic therapy change; thus, the competing risk model is unnecessary [15].

### Subgroup analyses

Subgroup analyses will be done within an exploratory framework. For the primary endpoint, the interaction term of the study group and stratification variables of randomisation will be explored. Estimated marginal intervention effects for each subgroup and 95%CI will be reported. The following subgroups will be analysed for possible differential intervention effects:

- Metastasis (secondary/therapy received, primary/therapy naïve)
- Tumour subtype (HR + /HER2 + , HR + /HER2 −, HR −/HER2 + , TNBC)
- Site of metastasis (cerebral, visceral, osseous, multiple)
- Age at randomisation (< 50 and ≥ 50)
- Types of systemic therapy at randomisation (endocrine, chemotherapy, anti-Her2 therapy, immunotherapy, targeted therapy, and others)
- Relationship status (partnership/marriage, single)
- Income (median split)
- Depression (yes/formerly, no)
- Body mass index (BMI) (< 30, ≥ 30 kg/m^2^)
- Changing in alarm criteria (before and after September 1, 2022)

Subgroup analyses for OS between intervention and control group will be performed separately for the patients with visceral metastases and the patients with triple-negative breast cancer.

### Loss of follow-up and missing data

**Fig. 2:**
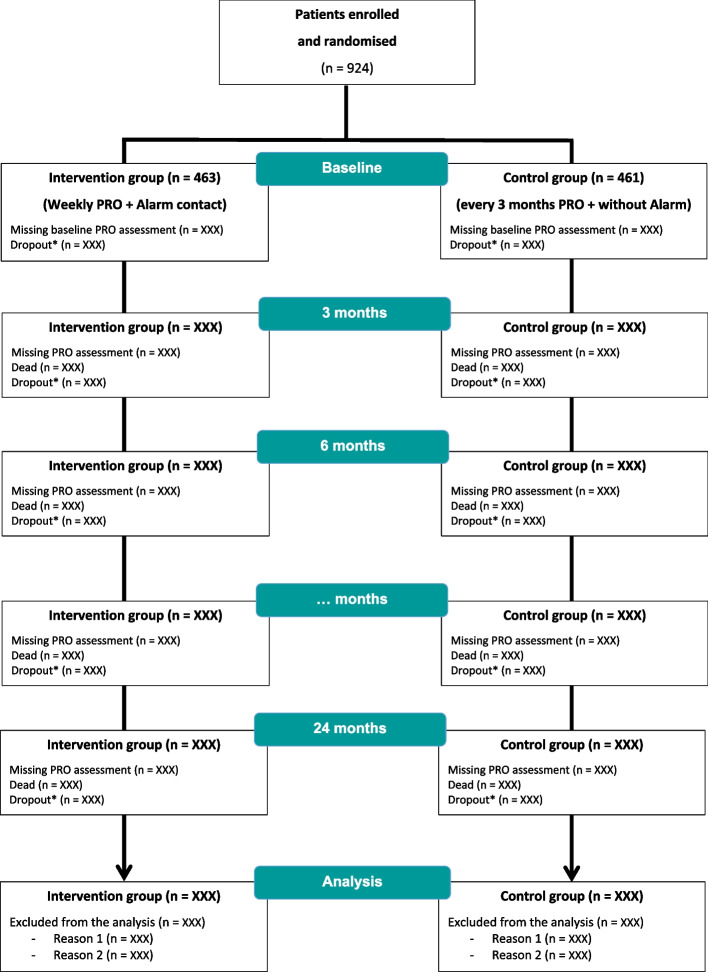
An example of PRO B study consort diagram

We will present the numbers of loss to follow-up, discontinuation, and death between the study groups and each follow-up time point in the trial flow diagram (Fig. 2). Patients will be deemed lost to follow-up if they no longer respond to the PRO surveys until the study ends. The last date of response to the survey will be considered the date of the last follow-up. If patients do not respond to the PRO survey at any given time point, but they return to answer the PRO survey in the following survey, the PRO missing data will be imputed and considered as missing at random (MAR). Discontinuation with reasons is documented and will be presented as percentages. The worst-case scenario will be applied to the missing PRO survey due to patients being too ill or if the intercurrent event of premature death happens [16]. Handling missing data is based on an estimand framework suggested by the International Council for Harmonisation of Technical Requirements for Pharmaceuticals for Human Use (ICH) guidance [17]. Table 2 presents the analysis plan for PRO B study data based on an estimand framework for our primary outcome. Patients who die or discontinue due to illness before the measurement time point will be accounted for in the PRO values using the worst-case scenario.

**Table 2:** An estimand framework for estimating the intervention effect in the analysis of the primary outcome in the PRO B study

**Scientific research question**	**Is the average in fatigue score measured by the EORTC CAT Core after** 6** months lower (superior) in the intervention group compared to the control group?**
**Estimand attributes**	
Intervention	Intervention: patients receiving weekly PRO surveys with an alarm contact in case of worsening PRO scores Control: patients receiving quarterly PRO surveys without an alarm contact in case of worsening PRO scores
Target population	**-** Female with MBC **-** Older than 18 years **-** Able to read and understand German **-** Receive drug treatment for MBC with a life expectancy at enrolment of more than 3 months **-** Having access to the Internet through a smartphone **-** Having the ECOG performance status of 0 to 2
Endpoint of interest	Fatigue score at 6 months post-randomisation
**Addressing intercurrent events**	
Discontinuation	
- Overload due to the PRO survey	Assuming missing data at random, implicitly impute data using MICE with *m* = 30 imputations
- Lack of interest or relevance	Assuming missing data at random, implicitly impute data using MICE with *m* = 30 imputations
- Patients feel too ill to continue participating in the study	Assuming missing data is not at random, a worst-case scenario will be applied
Loss to follow-up	Assuming missing data at random, implicitly impute data using MICE with *m* = 30 imputations
Death	Assuming missing data is not at random, a worst-case scenario will be applied
**Estimator**	Linear mixed model, adjusting for baseline fatigue score and potential confounding factors^a^. The study centre is set as a random intercept
**Summary measure**	Mean difference of fatigue score (IG–CG) and 95%CI (two-sided)
**Interpretation**	The mean difference of the fatigue score and the upper 95%CI limit are less than 0, it means the intervention group is superior to the control group

*CG* control group, *IG* intervention group, *ECOG* Eastern Cooperative Oncology Group, *MBC* metastatic breast cancer, *MICE* multiple imputation by chained equations, *PRO* patient-reported outcome

^a^Potential confounding factors are listed in the statistical methods section

### Multiple Imputation (MI)

Missing data, assumed to be MAR or missing completely at random (MCAR), will be imputed using multiple imputation by chained equations (MICE). MICE will be performed based on predictive mean matching for continuous variables, ordinal logistic regression models for ordinal variables, and binary logistic regression models for binary variables. All potential confounding factors (as listed above) will be included in the imputation model, along with additional factors such as study centres and all outcomes. The imputation will be carried out separately for each randomised group [18], and 30 imputed datasets will be generated. The results will be pooled based on Rubin’s rules [19]. Please note that patients who are not fully observed for a long-term outcome, particularly regarding late recruitment, will not be considered as missing data, and no imputation will be applied.

### Sensitivity analysis

Sensitivity analysis will be performed to assess the robustness of the primary analysis result under the assumption of missing not at random (MNAR). This will involve using a reference-based sensitivity analysis and a delta-adjusting pattern-mixture approach for tipping point analysis (TPA) [20, 21]. The reference-based sensitivity analysis employs a multiple imputation approach for patients who drop out of the PRO monitoring in the intervention group. The missing values will be imputed in a manner similar to the imputation used for the control group [21]. TPA assumes a systematic difference between the conditional distributions of the missing and observed data. To account for this difference, a shift parameter $\delta$ will be applied to the imputation model when imputing the missing data points. For each value of $\delta$, multiple imputed datasets will be generated. Each dataset will be analysed using the same method as the primary analysis, and the results will be combined using Rubin’s rule [19]. By varying the values of *δ*, the impact of missing data on the analysis results will be examined to identify tipping points—values at which the conclusions change from being significant to being insignificant between study groups.

### Statistical software

The analyses will be conducted using Stata MP/18 (StataCorp, 2023, College Station, TX, USA). The used packages will be included in the final report.

### Data management

The clinical data and PRO data are provided in *json* format. We will use Python to convert the data from *json* format to *csv* format. All the data management will be done using Stata MP/18 (StataCorp, 2023, College Station, TX, USA).

## Conclusion

The PRO B study aims to explore the transferability of digital alarm-based PRO monitoring benefits into German routine care and its positive effects on HRQoL and overall survival in metastatic breast cancer patients. This paper provides details of the planned statistical analysis strategies, aiming to reduce the bias of reporting results, and serves as guidance to support statisticians dealing with the analysis of PRO-based interventions (version 1.0; January 18, 2024).

### Supplementary Information

**Supplementary Material 1.**

## Data Availability

Data sharing is not applicable to this article as no datasets were generated or analysed.

## Abbreviations

CAT: Computer Adaptive Testing
CI: Confidence interval
EORTC: European Organisation for Research and Treatment of Cancer
GCP: Good Clinical Practice
HER: Human epidermal growth factor receptor
HR: Hormone receptor
HRQoL: Health-related quality of life
mITT: Modified intention-to-treat
MAR: Missing at random
MBC: Metastatic breast cancer
MI: Multiple imputation
MAR: Missing at random
MCAR: Missing completely at random
MNAR: Missing not at random
PRO: Patient-reported outcome
SAP: Statistical analysis plan
SD: Standard deviation
TPA: Tipping point analysis
