# Supplementary material for: Statistical analysis plan for the PRO B study: open-label, superiority randomised controlled trial of alarm-based patient-reported outcome monitoring in patients with metastatic breast cancer
Source: Trials. 2024 Mar 7;25:171. doi: 10.1186/s13063-024-08025-9 (PMC10918931; doi:10.1186/s13063-024-08025-9)
Supplement: Supplementary file 1 — Supplementary Material 1. [file 13063_2024_8025_MOESM1_ESM.docx]

**Supplementary**

**Table S1** Example of presentation of the socio-economic and anamnesis baseline characteristics in PRO B study

| **Baseline characteristics** | **Total**  **(n = XXX)** | **Intervention group**  **(n = XXX)** | **Control**  **Group**  **(n = XXX)** |
| --- | --- | --- | --- |
| Age at randomisation (years) |  |  |  |
| Mean (SD) [Mininum, Maximum] |  |  |  |
| **Socio-economic variables** |  |  |  |
| Migration status (yes/no) | n (%) | n (%) | n (%) |
| Marital status | n (%) | n (%) | n (%) |
| Married/ living with partner |  |  |  |
| Married/ living separately with partner |  |  |  |
| Single |  |  |  |
| Divorced |  |  |  |
| Widowed |  |  |  |
| Education | n (%) | n (%) | n (%) |
| Low |  |  |  |
| Medium |  |  |  |
| High |  |  |  |
| Employment status | n (%) | n (%) | n (%) |
| Full-time |  |  |  |
| Part-time |  |  |  |
| Retire |  |  |  |
| Mini-job |  |  |  |
| Irregularly employed |  |  |  |
| In vocational training |  |  |  |
| Others |  |  |  |
| Number of persons in the household |  |  |  |
| Mean (SD) or Median [IQR] |  |  |  |
| Having dependents in need of care or underage children (≤14 years) in the household (yes/no) | n (%) | n (%) | n (%) |
| Incoming (multiple categories) | n (%) | n (%) | n (%) |
| **Anamnesis variables** |  |  |  |
| Menopause (yes/no) | n (%) | n (%) | n (%) |
| Comorbidities (yes/no) | n (%) | n (%) | n (%) |
| NCI comorbidity index – Mean (SD) |  |  |  |
| Previous operations (yes/no) | n (%) | n (%) | n (%) |
| ECOG (0/1/2/3/4) | n (%) | n (%) | n (%) |
| Alcohol (never/rarely/occasionally/weekly/daily) | n (%) | n (%) | n (%) |
| Smoking (no, ex-smoker, current) | n (%) | n (%) | n (%) |
| Family history of cancer (yes/no) | n (%) | n (%) | n (%) |
| Burden in answering the PRO survey (not at all, little, moderate, very much) | n (%) | n (%) | n (%) |
| Preference for paper-based PRO survey (yes/no) | n (%) | n (%) | n (%) |
| Feeling confident using a smartphone (yes/no) | n (%) | n (%) | n (%) |

NCI = National Cancer Institute
